# Experimentally Induced Reductions in Alcohol Consumption and Brain, Cognitive, and Clinical Outcomes in Older Persons With and Those Without HIV Infection (30-Day Challenge Study): Protocol for a Nonrandomized Clinical Trial

**DOI:** 10.2196/53684

**Published:** 2024-04-02

**Authors:** Robert L Cook, Veronica L Richards, Joseph M Gullett, Brenda D G Lerner, Zhi Zhou, Eric C Porges, Yan Wang, Christopher W Kahler, Nancy P Barnett, Zhigang Li, Suresh Pallikkuth, Emmanuel Thomas, Allan Rodriguez, Kendall J Bryant, Smita Ghare, Shirish Barve, Varan Govind, Jessy G Dévieux, Ronald A Cohen, The 30-Day Challenge Research Team

**Affiliations:** 1 Southern HIV and Alcohol Research Consortium University of Florida Gainesville, FL United States; 2 Edna Bennett Pierce Prevention Research Center The Pennsylvania State University University Park, PA United States; 3 Florida International University Miami, FL United States; 4 Center for Cognitive Aging and Memory University of Florida Gainesville, FL United States; 5 Department of Behavioral and Social Sciences Center for Alcohol and Addiction Studies Brown University School of Public Health Providence, RI United States; 6 Miami Center for AIDS Research University of Miami Miller School of Medicine Miami, FL United States; 7 National Institute on Alcohol Abuse and Alcoholism Bethesda, MD United States; 8 Department of Medicine University of Louisville Louisville, KY United States; 9 See Acknowledgments Gainesville, FL United States

**Keywords:** alcohol, contingency management, biosensor, HIV infection, cognitive function

## Abstract

**Background:**

Both alcohol consumption and HIV infection are associated with worse brain, cognitive, and clinical outcomes in older adults. However, the extent to which brain and cognitive dysfunction is reversible with reduction or cessation of drinking is unknown.

**Objective:**

The 30-Day Challenge study was designed to determine whether reduction or cessation of drinking would be associated with improvements in cognition, reduction of systemic and brain inflammation, and improvement in HIV-related outcomes in adults with heavy drinking.

**Methods:**

The study design was a mechanistic experimental trial, in which all participants received an alcohol reduction intervention followed by repeated assessments of behavioral and clinical outcomes. Persons were eligible if they were 45 years of age or older, had weekly alcohol consumption of 21 or more drinks (men) or 14 or more drinks (women), and were not at high risk of alcohol withdrawal. After a baseline assessment, participants received an intervention consisting of contingency management (money for nondrinking days) for at least 30 days followed by a brief motivational interview. After this, participants could either resume drinking or not. Study questionnaires, neurocognitive assessments, neuroimaging, and blood, urine, and stool samples were collected at baseline, 30 days, 90 days, and 1 year after enrollment.

**Results:**

We enrolled 57 persons with heavy drinking who initiated the contingency management protocol (mean age 56 years, SD 4.6 years; 63%, n=36 male, 77%, n=44 Black, and 58%, n=33 people with HIV) of whom 50 completed 30-day follow-up and 43 the 90-day follow-up. The planned study procedures were interrupted and modified due to the COVID-19 pandemic of 2020-2021.

**Conclusions:**

This was the first study seeking to assess changes in brain (neuroimaging) and cognition after alcohol intervention in nontreatment-seeking people with HIV together with people without HIV as controls. Study design strengths, limitations, and lessons for future study design considerations are discussed. Planned analyses are in progress, after which deidentified study data will be available for sharing.

**Trial Registration:**

ClinicalTrials.gov NCT03353701; https://clinicaltrials.gov/study/NCT03353701

**International Registered Report Identifier (IRRID):**

DERR1-10.2196/53684

## Introduction

Alcohol misuse, or drinking in a manner that could cause harm to the user or those around them [1], is associated with poor HIV-related health outcomes (eg, lower rates of HIV viral suppression and suboptimal adherence to antiretroviral therapy) [2,3]. Alcohol consumption can contribute to a multitude of additional deleterious health effects, such as reductions in brain functioning, cognitive decline, liver disease, and systemic inflammation [4-8]. People with HIV tend to experience worse outcomes, including all-cause mortality, associated with alcohol misuse compared to persons without HIV [9]. Of additional concern is the combination of alcohol misuse and aging among people with HIV, as approximately 58% of people with HIV in the United States are at least 50 years of age [10]. Even mild cognitive impairments have detrimental functional effects and health outcomes that worsen as people with HIV age.

**Figure 1:**
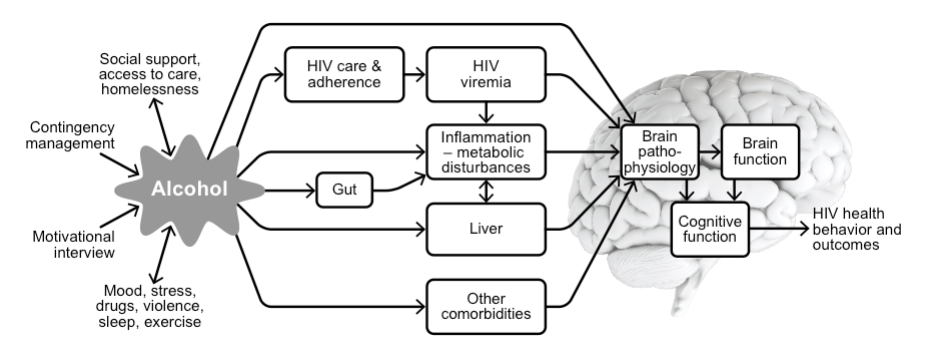
Conceptual model of the possible mechanisms by which alcohol could influence HIV-related health outcomes via the gut-brain axis.

Several mechanisms have been proposed to explain how alcohol consumption could impact chronic disease outcomes. One of the most common is related to the gut-liver-brain axis (see Figure 1), and proposes that alcohol consumption can result in both alterations of the gut microbiota (dysbiosis) as well as microbial translocation, with resulting systemic inflammation that then impacts the liver and brain [11]. While several studies have shown chronic alcohol use to be associated with negative cognitive effects, if any current cognitive effects are due to current brain inflammation, then a reduction in drinking could result in a reduction in inflammation and improved cognition. However, the extent to which these cognitive effects are reversible versus permanent is not known. Since people with HIV also have increased rates of cognitive decline and chronic systemic inflammation, they may be especially vulnerable to the impact of alcohol and might benefit the most from alcohol reduction.

The true causal effect of alcohol consumption on these health outcomes can be difficult to tease out with observational studies. While it is not ethical to challenge persons to engage in ongoing heavy drinking to determine its adverse effects, removing alcohol consumption from heavy drinkers could help to identify whether certain health aspects are reversible when drinking is removed. Therefore, the research team designed a study to determine whether cessation of drinking or significant reduction would be associated with improved brain function, cognition, and HIV clinical outcomes. The study design would also examine whether these outcomes got worse again with any resumption of drinking and would examine potential biological mechanisms related to gut microbial dysbiosis, intestinal permeability, biomarkers of systemic inflammation, liver function, and brain pathology.

In order to experimentally reduce drinking, the research team proposed contingency management (CM), a well-established intervention for treating alcohol use disorder [12,13] that provides financial payments to encourage individuals to abstain from alcohol use [14]. In order to monitor daily drinking status, the research team used transdermal alcohol sensors, because these can continuously and noninvasively monitor alcohol use [15,16]. Specifically, the Secure Continuous Remote Alcohol Monitor Continuous Alcohol Monitoring (SCRAM CAM; Alcohol Monitoring Systems, Inc), a sensor locked onto the ankle, has effectively been used in CM trials [12,17-20]. The sensor data can be used to determine whether reinforcement should be provided and can be used as an objective evidence of reduction. Motivational interviewing (MI) is another intervention with a demonstrated ability to reduce alcohol consumption [21,22] and was proposed as a booster intervention to help maintain alcohol reduction after CM was removed. The purpose of this study was not to evaluate the effectiveness of either CM or MI as alcohol interventions (since these are already known to be effective), but rather to use the interventions as a method of experimental manipulation to produce maximal drinking reduction in the short term (30-90 days) and provide objective verification of that reduction in order to study the effects of these changes in drinking on the body.

The primary aims of the study were to determine whether CM-induced alcohol reduction would improve cognitive performance and brain function among people with HIV in as little as 30 days and up to 1 year. If the impact of alcohol on systemic and cerebral inflammation is temporary, then reducing or eliminating alcohol consumption could dramatically improve cognitive function and indices of brain health, even among people who have consumed alcohol for many years. We sought to focus the research on people with HIV, because the potential benefits from alcohol cessation may be even greater due to the interactive effects. A smaller group of persons without HIV was included as a control population. Secondary aims were (1) to determine the impact of alcohol reduction on HIV clinical status, markers of systemic inflammation, and liver fat and fibrosis; (2) to investigate factors associated with success in reducing or stopping drinking; (3) to identify mechanisms linking drinking changes to HIV-related behavior and clinical outcomes; and (4) to identify the optimal measures of individual alcohol consumption using biosensors, biomarkers, and self-report.

## Methods

### Study Design and Overview

This was a nonrandomized, single-arm clinical trial that used a pre-post comparison with extended follow-up. The majority of participants would be people with HIV but a subset of persons without HIV were also included as controls and to allow for subgroup comparisons. After confirming eligibility, all participants provided preintervention data and then received a CM intervention to stop or reduce drinking for 30 to 90 days, and a MI intervention 30 days after baseline. Detailed clinical and behavioral assessments were collected at baseline, 30 days, 90 days, and 12 months after enrollment. The first participant was enrolled in December 2017 and final participant data were collected in April 2022. The study procedures were modified after study initiation to include additional data related to the gut microbiome and to adapt to the COVID-19 pandemic in 2020-2022.

### Ethical Considerations

Ethics approval was obtained from the institutional review boards at Florida International University (FIU; IRBSITE00000291), the University of Florida (UF; CED000000011), and the University of Miami (UM, 20170396). The study was registered at ClinicalTrials.gov (NCT03353701) in November 2017.

### Recruitment and Informed Consent

Participants were recruited in the Miami Metropolitan region (Florida) from HIV clinics, community outreach, and a contract registry. Recruitment advertisements were placed on public transportation and in local HIV clinics. Potential participants were screened over the phone or in person and those initially eligible were invited to attend an enrollment visit to review procedures, obtain consent, and confirm eligibility.

### Enrollment Visit

Potential participants were informed that one of the major goals of the study was to examine changes in the body after drinking reduction, and therefore they were asked to participate in a “30-Day Challenge,” in which they would try to reduce or stop drinking for at least 30 days. After informed consent was obtained, participants completed a detailed assessment to determine whether they were eligible to continue with the study.

The inclusion criteria were age 45-75 years, 21 or more drinks per week for men or 14 or more drinks per week for women, confirmed HIV status (for those who reported being HIV positive), English speaking, willingness to participate in CM, and wear an alcohol biosensor for at least 30 days. Exclusion criteria included neurological disorders (eg, dementia, stroke, seizures, and traumatic brain injury); past opportunistic infection; major psychiatric disturbance (eg, severe major depression); unstable medical conditions (eg, cancer); magnetic resonance imaging (MRI) contraindications (eg, pregnancy, severe claustrophobia, metal implants, and physical impairment precluding motor response or lying still); inability to demonstrate an understanding of key aspects of the study; and currently participating in other alcohol research.

Additional assessments used to determine eligibility at this enrollment visit included the Montreal Cognitive Assessment (MoCA) [23], the Alcohol Withdrawal Symptom Checklist (AWSC) [24], and a 30-Day Drug and Alcohol Timeline Followback (TLFB) [25]. Participants with MoCA scores lower than 17 were discussed with the investigators and allowed to proceed if they could clearly discuss the study goals and purpose with the research assistants. Those with AWSC scores greater than 8 were excluded due to the high risk of alcohol withdrawal. The TLFB was used to determine the average number of drinks per week, and persons with less than 21 drinks per week (men) or 14 drinks per week (women) were also excluded from the clinical intervention at this point.

### Pre-CM Test Week

At the enrollment visit, those who appeared to be eligible had the SCRAM CAM biosensor placed on their ankle. The monitor strap has a specialized clip that prevents removal without breaking the clip or cutting the ankle strap (and any removal sends an alert to SCRAM Systems and is viewable by the research team). The purpose of participants wearing the monitor for this week was to confirm that they did drink (for at least 3 days), they could go at least 24 hours without drinking and without withdrawal symptoms, they could tolerate wearing the SCRAM CAM, and they would communicate as expected with the research assistant. During this pre-CM phase, participants were instructed to drink as they normally would, except for at least 1 day of required abstinence (for persons who drink every day). Participants were given instructions about the monitor, including not submerging the device in water, avoiding using alcohol-based items, and not wearing socks under the monitor. A research assistant called the participant every other day to collect information on self-reported drinking and compared this information to that obtained by remote download from a cloud server from the SCRAM website. This ensured that for each participant, the monitor could accurately distinguish drinking from nondrinking days. During this pre-CM test week, participants received incentive payments for providing self-reports but no incentive for drinking behavior itself. During this pre-CM test week, some participants chose to withdraw, mostly due to not wanting to wear the ankle biosensor, and some were excluded because they did not drink enough or were not able to follow study procedures. All other people were scheduled to attend an in-person baseline assessment and to choose a specific date on which they would start the 30-Day Challenge.

### Baseline Assessment

The baseline assessment included a study questionnaire, neurocognitive assessments, neuroimaging assessments, liver Fibroscan, and collection of blood, urine, and stool samples. The baseline questionnaire was completed either during the enrollment visit (before the test week) or at the baseline assessment. Participants were required to have a 0 breathalyzer reading in order to proceed with the baseline questionnaire. The primary domains of the measures and the administration schedule are in Table 1. The study questionnaire items are available from the research team upon request.

**Table 1:** Summary of items assessed by study questionnaires for the 30-Day Challenge study at baseline and follow-up visits.

Domain (source)			Baseline		30 days		90 days		1 year
**Sociodemographics**									
	Age, country of origin, race or ethnicity, sex at birth, gender, sexual orientation, education, and incarceration history	✓							
	Marital status, homelessness, insurance, employment, income, and disability	✓						✓	
**Quality of life**									
	Quality of life (SF-12^a^ Health Survey v1) [26]	✓		✓		✓		✓	
	Physical activity (Godin-Shephard Leisure-Time Physical Activity Questionnaire) [27]	✓		✓		✓		✓	
**HIV care and medical history**									
	HIV/AIDS status, ART^b^ treatment adherence, and drinking impact on medication adherence	✓		✓		✓		✓	
	Year of first HIV positive test and HIV medications	✓							
	Self-reported medical conditions and current medications	✓							
	COVID-19 diagnosis history and vaccination status	✓							
**Symptoms**									
	General symptoms (Veterans Aging Cohort Study Survey) [28]	✓		✓		✓		✓	
	Sleep quality-2 items (Pittsburgh Sleep Quality Assessment) [29]	✓		✓		✓		✓	
	Pain (Brief Pain Inventory Short Form) [30] and self-reported current and past pain treatments	✓		✓		✓		✓	
	Alcohol use for pain and effectiveness of alcohol use for pain	✓							
**Mental health**									
	Anxiety (Generalized Anxiety Disorder-7) [31]	✓		✓		✓		✓	
	Depression (Patient Health Questionnaire-8) [32]	✓		✓		✓		✓	
	Posttraumatic stress disorder (PTSD; Primary Care PTSD Screen) [33]	✓		✓		✓		✓	
	Emotion regulation (difficulties in emotional regulation scale) [34]	✓				✓			
	Childhood trauma 4 items (Childhood Traumatic Events Scale) [35]	✓							
	Self-reported cognitive functioning (MOS^c^ Mental Health) [36]	✓		✓		✓		✓	
**Substance use**									
	30-day alcohol and drug use timeline follow-back [25]	✓		✓		✓		✓	
	AUDIT-C^d^ [37]	✓						✓	
	Alcohol use disorder assessment DSMV^e^ Alcohol Assessment [38]	✓						✓	
	Drinking motives (Drinking Motive Questionnaire) [39]	✓							
	Alcohol use of important persons (3 items), age of drinking onset, previous alcohol treatment, and expectancies about quitting drinking	✓							
	Open-ended questions about expectancies and outcomes related to the 30-day challenge and any changes in drinking	✓		✓		✓			
	Drug use frequency, including tobacco, readiness to quit smoking, injection drug use, noninjection drug use (Medical Monitoring Project survey) [40]	✓		✓		✓		✓	
	Lifetime alcohol use and alcohol drinking related to COVID-19 (adapted version of KMSK)^f,g,h^ [41]	✓							
**Sexual history**									
	Sexual behaviors (VACS^i^ Patient Survey) [28] and substance use before sex	✓		✓		✓		✓	
	Sexual function and satisfaction—4 items (PROMIS^j^ sexual function and satisfaction measures) [42]	✓		✓		✓		✓	

^a^SF-12: Short Form Health Survey.

^b^ART: antiretroviral treatment.

^c^MOS: medical outcomes study.

^d^AUDIT-C: Alcohol Use Disorders Identification Test for Consumption.

^e^DSMV: Diagnostic and Statistical Manual-V.

^f^KMSK: Kreek-McHugh-Schluger-Kellogg.

^g^Newly added to the study in revisions.

^h^Administered at other timepoints if baseline completed.

^i^VACS: Veterans Aging Cohort Study.

^j^PROMIS: Patient-Reported Outcomes Measurement Information System.

### Neurocognitive Assessments

A comprehensive battery of neuropsychological measures was administered to all participants (Table 2). The National Institutes of Health (NIH) toolbox (cognition battery) was given to participants to obtain an estimate of their crystalized (2 assessments) and fluid intellect (5 assessments) [43]. Uncorrected summary scores were created for the NIH toolbox crystalized and fluid scores and further analyses of these index scores included demographic factors such as age, race, and education in the models. For follow-up cognitive assessments, we used different forms when available, because this helps reduce the magnitude of practice effects.

Due to reported participant fatigue in the early phases of the research, the initial battery was refined to be completed in 1 hour or less (we dropped the California Computerized Assessment Package [CALCAP], Wechsler Adult Intelligence Scale-Fourth Edition [WAIS-IV], Brief Visuospatial Memory Test-Revised [BVMT-R]). Research assistants met with the team neuropsychologist regularly to ensure the best practices to maintain rapport and participant engagement. Reminders were given that the tests were purposefully created to be difficult, breaks were offered when somnolence was observed, and the research staff documented when people appeared to be providing limited effort. A summary of the specific neurocognitive tests and neuroimaging assessments obtained at their respective timepoints is included in Table 2.

For data analysis, the primary outcome for neurocognitive assessments is the change in performance on the 5 measures comprising the Fluid Cognition index from the NIH toolbox, which includes memory, attention, cognitive flexibility, processing speed, and executive functioning. Secondary outcomes of cognition will include changes in the other neuropsychological measures.

**Table 2:** Battery of neurocognitive tests for participants in the 30-Day Challenge study^a^.

Test name	Comments	Cognitive domain
NIH^b^ toolbox—Fluid [43]	Dimensional change card sort Flanker inhibitory control and attention Picture sequence memory List sorting Pattern comparison	Executive function Executive function and attention Episodic memory Working memory Processing speed
NIH toolbox—Crystalized^c^ [43]	Picture vocab Oral reading recognition	Premorbid intellect
Montreal Cognitive Assessment^c^ [23]	N/A^d^	General screen cognitive function
Trail Makings Test, Part A [44]	Cognitive flexibility	Graphomotor processing speed
Trail Makings Test, Part B [44]	N/A	Graphomotor processing speed and executive function
Stroop Test [45]	Cognitive flexibility	General processing speed and inhibitory function
Hopkins Verbal Learning Test-Revised (HVLT-R) [46]	Included 3 learning trials, a delayed recall, and a recognition trial	Verbal learning and memory
Wechsler Adult Intelligence Scale-Fourth Edition (WAIS-IV) [47]	Symbol search Digit span Letter number sequencing	Graphomotor processing speed Auditory attention Working memory
Adaptive Rate Continuous Performance Test (ARCPT)^e^ [48]	N/A	Maintained attention and inhibitory control
Controlled Oral Word Association Test [49]	FAS or CFL^f^	Language function, processing speed, and verbal fluency
Animal Fluency [50]	N/A	Semantic verbal fluency
Card Sorting Task^g^ [51]	N/A	Novel problem-solving and set-shifting
Grooved Pegboard Test [52]	N/A	Fine motor dexterity

^a^Unless otherwise indicated, assessments were done at baseline, 30 days, 90 days, and 1 year after enrollment.

^b^NIH: National Institutes of Health.

^c^The Montreal Cognitive Assessment and the NIH Crystalized measures were only obtained at baseline.

^d^N/A: not applicable.

^e^Some participants did the California Computerized Assessment Package (CALCAP) at baseline and then switched to Adaptive Rate Continuous Performance Test at follow-ups (30 did the Adaptive Rate Continuous Performance Test at baseline).

^f^CFL: Measure of spontaneous production of words beginning with 3 letters (FAS or CFL).

^g^The Wisconsin Card Sorting Task was collected as part of the COVID-19 supplement and was completed by 20 participants.

### Neuroimaging Assessments

Participants who had no contraindications underwent MRI neuroimaging at all 4 time points. Prior to the first MRI, participants were provided with instructions on the 2 functional MRI tasks. For example, they were administered the 2-back test, which required participants to view alphabets in English (ie, a stimulus) on a computer screen and indicate by clicking a button on a device held in their hand whether the currently displayed letter was the same or different from the letter that appeared 2 preceding times ago (ie, 2-back). The MRI protocol used for all 4 timepoints of this study is shown in Table 3. The total time to complete all the sequences in the MRI protocol was approximately 65 minutes.

**Table 3:** Neuroimaging protocol used at all 4 timepoints for participants in the 30-Day Challenge study.

Sequence	Purpose	Measure	Anatomical coverage
T1^a^ MRI^b^	To measure brain morphometric changes	Anatomical and tissue volumes and cortical thickness	Whole-brain
FLAIR^c^ MRI	Identification of incidental brain pathologies	Pathology type and its volume	Whole-brain
MEGA-PRESS^d^ Single-voxel MRS^e^ [53]	Quantitation of changes in GABA^f^ and other brain metabolites	Concentration of GABA, NAA^g^, Cre^h^, Cho^i^, and m-Ins^j^, and their ratios with Cre	Single voxel at the anterior mid-cingulate gyrus
2-back task-based fMRI^k^	Working memory	2-back alphabet letters	Whole-brain
Resting state fMRI	For assessment of changes in neural networks involved in the brain functional-segregation and functional-integration [54]	Functional connectivity measures for 5 major brain networks	Whole-brain
Diffusion tensor and kurtosis imaging [55]	To evaluate tissue microstructural changes	Diffusivities (axial, radial, and mean); fractional anisotropy (FA); free water fraction; kurtoses (axial, radial, and mean); kurtosis FA	Whole-brain
Whole-brain proton MR^l^ spectroscopic imaging [56]	Quantitation of changes in NAA, Cre, Cho, and m-Ins metabolites	Concentration of NAA, Cre, Cho, and m-Ins, and their ratios with Cre	Whole-brain

^a^T1: spin-lattice relaxation time.

^b^MRI: magnetic resonance imaging.

^c^FLAIR: fluid attenuated inversion recovery.

^d^MEGA-PRESS: Meshcher-Garwood Point Resolved Spectroscopy.

^e^MRS: magnetic resonance spectroscopy.

^f^GABA: γ-aminobutyric acid.

^g^NAA: N-acetyl aspartate.

^h^Cre: total creatine.

^i^Cho: total choline.

^j^m-Ins: myo-inositol.

^k^fMRI: functional magnetic resonance imaging.

^l^MR: magnetic resonance.

The primary outcome from neuroimaging assessments is changes in brain inflammation from baseline to subsequent timepoints at the regional, tissue-type (ie, gray matter and white matter), and whole-brain levels. This outcome will be assessed from the quantitation of cerebral metabolite markers of neuroinflammation (ie, total choline and myo-inositol) and extracellular free water fraction (a measure determined from diffusion tensor imaging data). The above neuroinflammation markers will be quantified from the brain regions including the basal ganglia, thalamus, and frontal lobe that are primarily involved in HIV infection, alcohol use disorders, and their interaction [57-59]. Changes in brain function from functional magnetic resonance imaging and resting-state connectivity data will be measured for 5 major networks, that is, the default mode network, the dorsal attention network, the salience network, the limbic network, and the fronto-parietal control network.

### Blood and Urine Testing

Blood samples were collected at each timepoint. Part of these were sent to a commercial laboratory for measurements of complete blood count with differential, comprehensive metabolic panel, hepatitis C antibody, HIV antibody (to confirm HIV status), HIV-1 RNA (only for people with HIV), and CD4 lymphocyte count (only for people with HIV). A dried blood spot was collected to measure phosphatidylethanol, an alcohol biomarker. We used these samples and performed measurements of cytokines, inflammatory biomarkers, adhesion molecules, and markers of intestinal permeability and microbial translocation. Additional blood samples are stored in a biorepository at the University of Louisville, where there is planned testing related to gut microbiome and gut-derived metabolites (metabolomics). Urine tests were performed at each timepoint for ethyl glucuronide (an alcohol biomarker), drug screen, and (after approximately 1 year) urine specific gravity. Urine-specific gravity was collected to ensure any potential changes in free water–based neuroinflammation (Table 4) were not solely due to brain rehydration after abstinence from alcohol.

**Table 4:** Summary of additional laboratory and clinical assessments conducted during the 30-Day Challenge study^a^.

Domain	Specific measures
Blood	All participants: complete blood count, comprehensive metabolic panel, phosphatidylethanol (alcohol biomarker), hepatitis C antibody (once), HCV^b^ viral load (tested in 2/5 who were HCV antibody positive). HIV-related: HIV antibody (for those who self-reported HIV-negative). HIV viral load and CD4 lymphocyte count (for HIV-positive). Cytokines and biomarkers of inflammation: TNF-RII^c^, TNFα^d^, IL-6^e^, IL-10^f^, IFN-γ^g^ NFL^h^, sCD163^i^, VCAM-1, ICAM-1, sCD14, CRP^j^, and LBP^k^. COVID-19^l^: RBD^m^ IgG^n^ and nucleocapsid IgG (in those RBD ab positive).
Urine	Drug screen (cocaine, methamphetamine, THC^o^, MDMA^p^, opioid, oxycodone, PCP^q^, barbiturates, and benzodiazepines). Specific gravity. Urine ethyl glucuronide (at each visit and to confirm self-reported abstinence with positive alcohol biosensor).
Stool	Gut microbiome: 16S rRNA^r^ gene sequencing, relative abundance, Firmicutes/Bacteroidota ratio (F/B)
Fibroscan	Controlled attenuation parameter (fatty liver), liver stiffness measurement (fibrosis and stiffness).

^a^Unless otherwise indicated, assessments were done at each timepoint.

^b^HCV: hepatitis C virus.

^c^TNF-RII: tumor necrosis factor receptor 2.

^d^TNFα: tumor necrosis factor α.

^e^IL-6: interleukin 6.

^f^IL-10: interleukin 10.

^g^IFNγ: interferon-γ.

^h^NFL: neurofilament light chain.

^i^sCD163: soluble CD163.

^j^CRP: C-reactive protein.

^k^LBP: lipopolysaccharide-binding protein.

^l^COVID-19 antibodies were tested on all blood samples obtained after March 1, 2020.

^m^RBD: receptor-binding domain.

^n^IgG: immunoglobulin G.

^o^THC: tetrahydrocannabinol.

^p^MDMA: methylenedioxymethamphetamine.

^q^PCP: phencyclidine.

^r^16S rRNA: 16S ribosomal RNA (or 16S ribosomal ribonucleic acid).

### Stool Samples for Gut Microbiome Assessments (Metagenomic Analysis)

During the first year of the study, the research team received additional funding to add a collection of stool samples for gut microbiome assessment and to measure additional blood biomarkers related to systemic inflammation (U01AA026225). These assessments, together with a food frequency questionnaire [60], were added after 17 participants had been enrolled, but 11 provided this information at a follow-up visit. Stool samples were sent to the University of Louisville for 16S rRNA gene sequencing processing, taxonomic evaluation, and determination of longitudinal changes in bacterial composition and diversity.

A Fibroscan liver test was obtained on all participants (Fibroscan 502 Touch, EchoSens, Paris, and France with the XL probe). The 2 scores were calculated using an average of 10 assessments, fat or controlled attenuation parameter score and fibrosis or liver stiffness measurement.

### Alcohol Interventions

#### Contingency Management

The CM period began after the completion of the baseline assessment. Initially, we sought to maintain abstinence for up to 90 days using CM payments based on reports from the ankle biosensor. After approximately 10 participants enrolled, we modified the protocol to include CM payments and ankle monitoring for only 30 days because the participants did not like to wear the SCRAM, and because the costs for both participant payments and SCRAM monitoring would exceed the awarded budget. The payment protocol followed recommended CM methods as well as prior research using the SCRAM [12,15] and incorporated information obtained from focus groups prior to initiating the study [61]. Participants would receive money for each day they were abstinent and additional bonus payments for completing 7 days of abstinence in a row. Abstinence was determined on a daily basis through ankle biosensor reports with payment amounts increasing for consecutive days. Specifically, on the first day of abstinence, a participant would receive US $5. For each consecutive day of abstinence thereafter (up to 7 in a row), the daily payout increased by US $1, meaning that after 7 days of abstinence, participants received a total of US $56 in daily payments plus a bonus of US $25 for maintaining abstinence for 7 consecutive days. Bonus payments increased by US $20 every 7 days up to a maximum weekly bonus of US $85. Participants received US $0 on any drinking day and the daily payment for abstinence restarted at US $5 after any drinking day. The maximum amount paid for maintaining abstinence throughout the CM period (30 days) was US $440. Participants were provided their payment as often as they wished but commonly chose to receive payments approximately once a week.

The alcohol biosensor provided data on transdermal alcohol concentration (TAC) assessed every 30 minutes. The specific TAC criteria used to differentiate a drinking day from a nondrinking day are based on several factors including peak TAC, absorption rate (rise rate), and fall rate (elimination rate). Different criteria can be used to minimize both false positives (for example, if alcohol is spilled on the device) and false negatives (for example, a participant may drink but not enough to reach a threshold level TAC). Our research team used the software (Transdermal Alcohol Sensor Data Macro [TASMAC] [62]) developed to identify drinking episodes on any given day (6 AM-6 AM), using more sensitive criteria than SCRAM Systems to detect drinking days (ie, peak TAC of at least 0.02 g/dL and either an absorption rate for the episode <0.05 g/dL per hour or an elimination rate for the episode <0.025 g/dL per hour [when peak <0.15 g/dL] and less than 0.035 g/dL per hour [when peak >0.15 g/dL]) [15]. If participants reported abstinence when alcohol was detected via the biosensor, the participants were given the opportunity to provide a negative in-person urine sample using dipstick ethyl glucuronide testing within 2 days of the SCRAM positive reading to maintain their CM payments.

#### Motivational Interview

During the 30-day visit, participants completed a single session of MI by videoconference using a computer within the clinical research setting. The MI was provided by a male, masters-level trained counselor at Brown University who had undergone over 20 hours of training in MI and had prior experience delivering MI. The MI session included a discussion of initial motivations for participating in the study, a review of the participant’s drinking behavior prior to and during the 30-Day Challenge, a discussion of the perceived benefits of changing drinking, steps taken to reduce drinking successfully during CM, and creation of a change plan, which included discussion of future goals around drinking and brief problem-solving around meeting those goals. The MI assessments took approximately 30-45 minutes and were recorded and transcribed for further analysis. Every other week, a clinical supervisor with over 10 years of experience supervising MI counselors would listen to a session, if available, and provide feedback on MI counseling skills.

### Follow-Up

#### Safety and Fidelity Monitoring

Several safety monitoring procedures were included in the protocol. A research assistant contacted participants on the first 3 days of CM to monitor for alcohol withdrawal symptoms. Research staff also collected self-reported data on drinking several times a week and helped with adjustments of the SCRAM monitor for comfort when needed. A study physician reviewed all laboratory results and participants were notified and referred to their physician for the occasional clinically significant laboratory finding. Potential adverse events were discussed with the research team and study principal investigators on a regular basis. During the first 3 years of the study, if research assistants noted anything that looked suspicious on neuroimaging, a radiologist was consulted, and participants were provided with information to discuss with their physician. For post–COVID-19 assessments, a clinical neuroradiologist reviewed every scan for clinically significant findings. No participants experienced serious alcohol withdrawal, and 1 participant was referred to their physician due to an abnormality found on brain MRI. To monitor fidelity to the research protocol, all research staff completed training and demonstrated the ability to do each of the study assessments. A senior research coordinator from our central coordinating team in Gainesville provided site monitoring in Miami 2-3 times per year.

#### Follow-Up Visits

Participants returned for in-person assessments at 30 days, 90 days, and 1 year. At each timepoint, updated alcohol consumption data were obtained, and the majority of the baseline measures were repeated (see Tables 2-4). The SCRAM monitor was removed at the 30-day visit for most participants (n=47), whereas 10 participants wore if for 90 days. Stool samples for gut microbiome assessments were obtained at baseline (n=40), 30 days (n=36), 90 days (n=36), and 1 year (n=23).

#### Procedures to Enhance Follow-Up

The study research staff communicated with participants regularly during the first 30 days (during the CM period) and scheduled follow-up visits in advance. Reminder calls were made to enhance adherence to follow-ups, which included additional participant incentives. The window period around each follow-up included at least 1 week before and 2 weeks after the scheduled appointment period.

### Modifications After Study Initiation

#### Duration of CM

Prior to study initiation, the research team was encouraged by NIH peer grant reviewers and a scientific advisory board to extend the CM (and ankle biosensor monitoring) to 90 days, because it was not known whether any benefits of alcohol cessation or reduction would be fully achieved by 30 days or if a longer period would result in further improvements. A total of 10 of the initial participants chose to continue CM payments and ankle monitoring for 90 days, but many declined to continue monitoring and nearly every participant complained about some aspect of wearing the ankle biosensor. Also, extending the CM from 30 to 90 days added substantially to the cost of the study (participant payments and SCRAM monitoring fees), and with input from an external scientific advisory board, the research team modified the protocol to its original plan of CM payments only to 30 days.

#### COVID-19–Related Modifications

All in-person research activities were halted for 3 months starting in March 2020, with limited in-person data collection beginning again in June 2020, and MRI studies resuming in July 2020. Study procedures were modified to include remote data collection, and 3 participants provided follow-up data remotely during this period. An NIH COVID-19 funding supplement supported the collection of qualitative data from a subset of participants, additional post–COVID-19 study assessments for interested participants, pilot-testing of remote neurocognitive assessments, and the testing of participant blood samples for COVID-19 antibodies. The research team ultimately decided that remote neurocognitive assessments could not be directly compared to the same assessments conducted face-to-face, and thus neurocognitive and neuroimaging data are missing from some participants at timepoints early during the COVID-19 pandemic in 2020.

### Planned Data Analysis

#### Overview

For our primary exposure variable (change in alcohol consumption), the primary assessment will focus on the average self-reported number of drinks per week during the 4-weeks prior to each timepoint (baseline, 30 days, 90 days, and 1 year), and in changes in self-reported number of drinks per week. Secondary metrics of drinking at each time point will include the number of heavy drinking days (previous 30 days), and categorical definitions of drinking status based on current National Institute on Alcohol Abuse and Alcoholism (NIAAA)–recommended drinking amounts (heavy, mild or moderate drinking, and no drinking). We plan to validate the self-reported measures with other measures of alcohol consumption, especially the SCRAM TAC readings (only collected for 7 days preintervention through 30 days for most participants).

Cross-sectional analyses are planned to compare characteristics of persons at baseline (eg, people with HIV vs controls), and to determine the association of alcohol consumption and potential confounding variables with key outcomes including gut microbial dysbiosis, neurocognition, neuroinflammation, biomarkers of systemic inflammation, Fibroscan liver scores, and HIV-related outcomes.

Longitudinal analyses will assess the relationships between changes in drinking and changes in each of the main clinical outcomes. For each main outcome, we will consider potential confounding variables and whether those are fixed or changing over time. Baseline values will be controlled in the longitudinal analyses. Multiple testing will be adjusted by the false discovery rate approach [63]. Missing data that are considered to be missing at random will be handled by multiple imputation or EM algorithms. Missing data that are considered to be nonignorable missing or missing not at random will be handled with pattern-mixture models.

#### Sample Size

**Figure 2:**
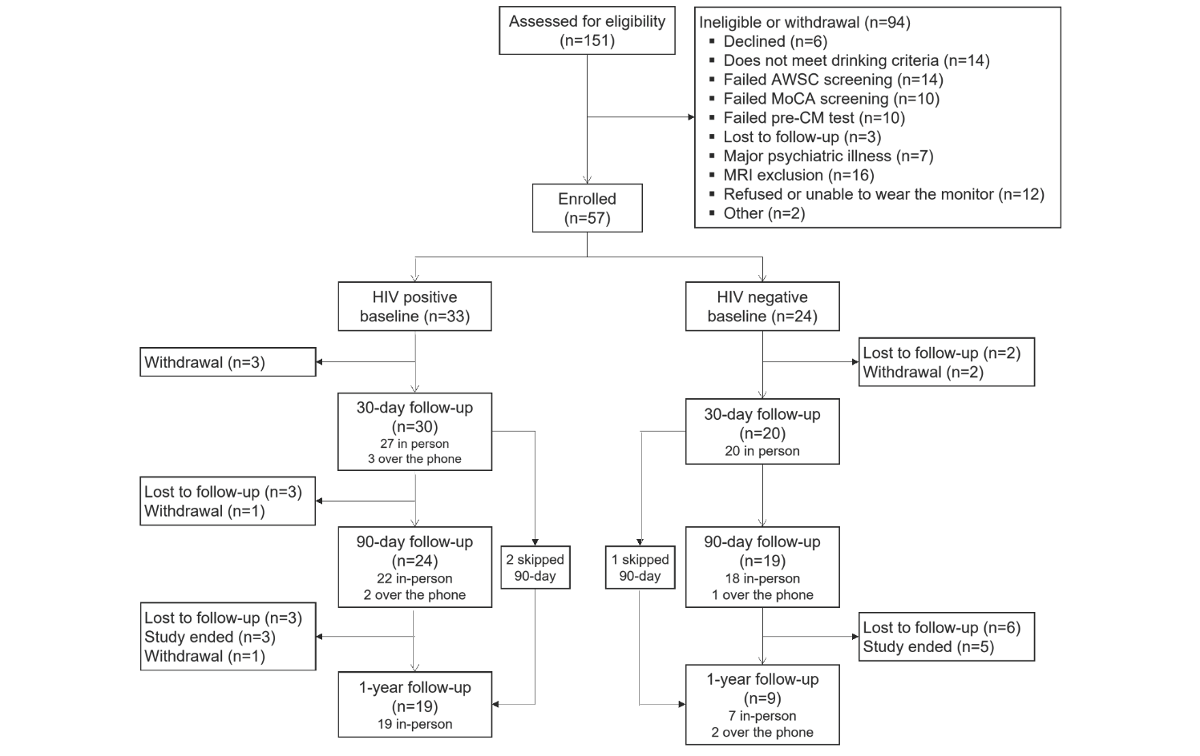
Flowchart of participants evaluated and enrolled in the 30-Day Challenge study. AWSC: Alcohol Withdrawal Symptom Checklist; CM: contingency management; MoCA: Montreal Cognitive Assessment; MRI: magnetic resonance imaging.

This study was originally approved to recruit 180 participants (140 people with HIV and 40 without HIV). However, the COVID-19 pandemic, availability of research staff and neuroimaging appointments, and reluctance of people to wear the ankle biosensor substantially impacted recruitment. Final enrollment numbers are detailed in Figure 2 and input from the scientific advisory board was obtained prior to cessation of enrollment and data collection.

## Results

Recruitment of participants began in December 2017 and ended in October 2021. Data collection was completed in April 2022. Baseline characteristics of 57 participants who initiated the 30-day challenge, including persons with and without HIV, are presented in Table 5. Of the 57 participants who initiated the 30-Day Challenge, 88% completed the 30-day follow-up, 75% completed the 90-day follow-up, and 49% completed the 1-year follow-up.

**Table 5:** Participant baseline characteristics in the 30-Day Challenge study (N=57).

Characteristics			Total (N=57)		Persons with HIV (n=33)		Persons without HIV (n=24)
**Gender, n (%)**							
	Man	36 (63)		19 (58)		17 (80)	
	Woman	20 (35)		13 (39)		7 (29)	
	Transgender	1 (2)		1 (3)		0	
**Age (years)**							
	Range	48-67		48-66		48-67	
	Mean (SD)	55.9 (4.6)		55.5 (4.3)		56.5 (5.1)	
**Race or ethnicity, n (%)**							
	Non-Hispanic, White	6 (10)		3 (9)		3 (12)	
	Non-Hispanic, Black	44 (77)		27 (82)		17 (71)	
	Hispanic	7 (12)		3 (9)		4 (17)	
**Marital status^a^, n (%)**							
	No	47 (82)		25 (76)		22 (92)	
	Yes	10 (18)		8 (24)		2 (8)	
**Education, n (%)**							
	Less than high school	18 (32)		13 (39)		5 (21)	
	High school graduate or GED	19 (33)		6 (18)		13 (54)	
	More than high school	20 (35)		14 (42)		6 (25)	
**Homeless in the past 12 months, n (%)**							
	No	50 (88)		29 (88)		21 (88)	
	Yes	7 (12)		4 (12)		3 (12)	
**Employment, n (%)**							
	Not employed	11 (19)		6 (18)		6 (21)	
	Employed	13 (23)		4 (12)		9 (38)	
	Unable to work or disabled	33 (58)		23 (70)		10 (42)	
**Anxiety^b^, n (%)**							
	None to minimal	29 (51)		15 (46)		14 (58)	
	Mild	17 (30)		11 (33)		6 (25)	
	Moderate	7 (12)		4 (12)		3 (12)	
	Severe	4 (7)		3 (9)		1 (4)	
**Depression^c^, n (%)**							
	None or minimal	30 (53)		18 (55)		12 (50)	
	Mild	16 (29)		8 (24)		8 (33)	
	Moderate	5 (9)		4 (12)		1 (4)	
	Moderately severe or severe	6 (10)		3 (9)		3 (12)	
**Alcohol use disorder^d^, n (%)**							
	No	5 (9)		4 (12)		1 (4)	
	Mild	6 (10)		3 (9)		3 (12)	
	Moderate	10 (18)		9 (27)		1 (4)	
	Severe	36 (63)		17 (51)		19 (79)	

^a^Married or living with a long-term partner.

^b^Measured by the Generalized anxiety disorder 7-item scale (GAD-7); scores of 5-9=mild, 10-14=moderate, and ≥15=severe.

^c^Measured by the Patient Health Questionnaire-8; scores of 0-4=none or minimal, 5-9=mild, 10-14=moderate, and ≥15=moderately severe or severe.

^d^Measured by diagnostic and statistical manual-V (DSMV) Alcohol Assessment; scores of 0-1=no, 2-3=mild, 4-5=moderate, and ≥6=severe.

## Discussion

Previous research on the impact of alcohol on HIV infection has been primarily observational, making it hard to determine whether outcomes associated with alcohol consumption are caused by the alcohol itself. We designed an experimental research study to obtain stronger evidence on whether changes, and specifically, reductions in drinking would correlate with changes in other behavioral or biological processes in the body. The study is unique from other research studies that examined changes in drinking and changes in clinical outcomes. Previous studies examining neurocognitive changes have primarily enrolled persons who were initiating alcohol treatment or included persons without HIV. Other strengths of the study include the simultaneous collection of a range of biological and behavioral data from several time points, including alcohol consumption, cognitive assessments, neuroimaging, Fibroscan liver test, blood biomarkers, and longitudinal changes in gut microbial dysbiosis.

Challenges in the study included recruitment, the complexity of research activity involving several universities and institutional review boards, staff turnover (and delays in replacing staff), participant willingness to wear the ankle biosensor, coordination of study procedures across several settings, and locations for persons who are often without their own transportation. Also, the number of assessments collected at each timepoint led to the decision to collect data over 2 days rather than 1, which limited our ability to enroll more participants. Future research teams should consider the tension between the value of having additional assessments versus the cost savings and convenience of collecting research study data over a single day (rather than spread across 2 days).

The COVID-19 pandemic, starting in early 2020, had a major impact on the study procedures and enrollment. A second clinical research setting was prepared to begin recruitment and data collection in early 2020, but by the time most research activities could be resumed after the COVID-19 pandemic, the study procedures had begun to wind down.

Another limitation of the study is that persons at high risk of alcohol withdrawal were excluded. The research team developed a protocol to enroll drinkers at higher risk of alcohol withdrawal but ultimately decided the risks and complexity outweighed the benefits.

## Appendix

Multimedia Appendix 1 Peer-review report from the National Institutes of Health.

## Abbreviations

AWSC: Alcohol Withdrawal Symptom Checklist
BVMT-R: Brief Visuospatial Memory Test-Revised
CALCAP: California Computerized Assessment Package
CM: contingency management
FIU: Florida International University
MI: motivational interviewing
MoCA: Montreal Cognitive Assessment
MRI: magnetic resonance imaging
NIAAA: National Institute on Alcohol Abuse and Alcoholism
NIH: National Institutes of Health
SCRAM CAM: Secure Continuous Remote Alcohol Monitor Continuous Alcohol Monitoring
TAC: transdermal alcohol concentration
TASMAC: Transdermal Alcohol Sensor Data Macro
TLFB: 30-Day Drug and Alcohol Timeline Followback
UF: University of Florida
UM: University of Miami
WAIS-IV: Wechsler Adult Intelligence Scale-Fourth Edition

## Data Availability

The data sets generated and analyzed during this study are available from the corresponding author on reasonable request.
