# Supplementary material for: Experimentally Induced Reductions in Alcohol Consumption and Brain, Cognitive, and Clinical Outcomes in Older Persons With and Those Without HIV Infection (30-Day Challenge Study): Protocol for a Nonrandomized Clinical Trial
Source: JMIR Res Protoc. 2024 Apr 2;13:e53684. doi: 10.2196/53684 (PMC11028398; doi:10.2196/53684)
Supplement: Multimedia Appendix 1 [file resprot_v13i1e53684_app1.pdf]

SUMMARY STATEMENT
(Privileged Communication)

PROGRAM CONTACT:
Dr. Joe Wang
301-451-0747
wangh4@mail.nih.gov

Release Date: 05/09/2016
Revised Date:

Application Number: 2 U01 AA020797-06

Principal Investigators (Listed Alphabetically):

COOK, ROBERT L (Contact)
DEVIEUX, JESSY G
GOVIND, VARAN

Applicant Organization: UNIVERSITY OF FLORIDA

Review Group: ZAA1 DD (10)
National Institute on Alcohol Abuse and Alcoholism Special Emphasis Panel
CHAART Consortium RFA
AIDS - EXP. REV.

Meeting Date: 04/27/2016
Council: MAY 2016
Requested Start: 07/01/2016
RFA/PA: AA16-001
PCC: AMAW

Project Title: Effects of experimentally-induced reductions in alcohol consumption on brain cognitive, and clinical outcomes and motivation for changing drinking in older persons with HIV infection
SRG Action: Impact Score:45
Next Steps: Visit http://grants.nih.gov/grants/next_steps.htm
Human Subjects: 30-Human subjects involved - Certified, no SRG concerns
Animal Subjects: 10-No live vertebrate animals involved for competing appl.
Gender: 1A-Both genders, scientifically acceptable
Minority: 1A-Minorities and non-minorities, scientifically acceptable
Children: 1A-Both Children and Adults, scientifically acceptable
Clinical Research - not NIH-defined Phase III Trial

Project Year	Direct Costs Requested	Estimated Total Cost
6	537,663	794,654
7	650,485	961,403
8	670,161	990,483
9	681,689	1,007,522
10	652,787	964,805
TOTAL	3,192,785	4,718,867

ADMINISTRATIVE BUDGET NOTE: The budget shown is the requested budget and has not been adjusted to reflect any recommendations made by reviewers. If an award is planned, the costs will be calculated by Institute grants management staff based on the recommendations outlined below in the COMMITTEE BUDGET RECOMMENDATIONS section.

2U01AA020797-06 Cook, Robert

RESUME AND SUMMARY OF DISCUSSION: This is a renewal collaborative U01 application submitted in response to RFA-AA-16-001 titled “Limited Competition: Consortia for HIV/AIDS and Alcohol–Related Research Trials (CHAART) Project” and proposes to investigate the effects of experimentally-induced reductions in alcohol consumption on brain & cognitive functions and clinical outcomes, as well as motivation for changing drinking in older persons with HIV infection. The investigators propose to enroll 180 heavy drinking HIV+ adults and use behavioral interventions using a combination of Contingency Management (CM) with financial incentives, continuous monitoring with wearable alcohol biosensors and motivational interviewing to maximally reduce alcohol consumption. The subjects will be assessed at baseline (T1), after 4-weeks of CM (T2), and a final assessment one year later (T3) for cognitive performance, brain functions and pathophysiology, drinking behavior, as well as HIV-associated health outcomes. The proposed studies are highly innovative and significant and could help clearly demonstrate whether alcohol effects on cognition and the brain are reversible, in addition to suggesting optimal strategies to promote short-term and long-term alcohol reduction in HIV+ adults. Additional strengths of the application include an outstanding and experienced team of investigators, as well as the excellent support and resources available through the administrative and research cores of Southern HIV Alcohol Research Consortium (SHARC). However, several methodological weaknesses reduce the significance and overall impact of this application. The proposed approaches are not supported by adequate preliminary data. The 4 weeks dose of contingency management (CM) period seems short, as most published trials have employed 12 and 16 weeks of in order to get a large number of participants to achieve abstinence. It is unclear from the pilot data, or previous literature, whether such a short period of potential abstinence could result in sustained cognitive benefits. The recruitment and data analysis plan are not provided in adequate detail; e.g., there is no apparent plan to handle missing data, and the justification for the expected dropout rate is lacking. Also, the analysis plan do not seem to address effects of partial reduction in drinking (as opposed to total abstinence), which may be a missed opportunity.

DESCRIPTION This proposed U01 study will build on our past findings to determine the extent to which marked reductions in alcohol consumption over 4-weeks via contingency management (CM) improves cognitive performance, brain functions and pathophysiology, and HIV-associated health outcomes. HIV-associated neurocognitive dysfunction continues even with antiretroviral treatment, and even mild cognitive impairment is associated with detrimental health outcomes in older HIV+ adults. Alcohol consumption may affect the brain directly or indirectly via liver toxicity and systemic inflammation. Our past findings indicate that current heavy alcohol use is more strongly associated with cognitive/brain dysfunction among HIV+ adults than lifetime consumption, suggesting that these effects may be reversible with reductions in drinking. Towards this objective, we propose to enroll 180 HIV+ adults with heavy drinking, and then use CM with financial incentives and a wearable alcohol biosensor to maximally reduce alcohol consumption from baseline (T1) to 4-weeks later (T2). We will then conduct a motivational interview to determine perceived benefits and obstacles to drinking reduction, and conduct a final assessment 1 year later (T3), at which point persons may or may not have resumed heavy drinking. We will conduct state-of-the-art neuroimaging, cognitive, and behavioral assessments at each time point, and then continue to track long-term drinking and HIV outcomes in our companion Cohort (U24). The Specific Aims of this proposal are: 1) to demonstrate improved cognitive performance and brain function (fMRI) after 4-weeks of CM-induced alcohol reduction among HIV+ adults, followed by worsening of these effects 1-year later if heavy drinking resumes; 2) to demonstrate that cerebral metabolic (MRS) and neuroinflammatory (DTI-free water) markers will also improve with CM-induced alcohol reduction and worsen if drinking resumes post-CM; and 3) Determine whether perceived benefits and challenges to drinking reduction identified during motivational interviewing (MI) predict drinking reductions or relapse one-year post-CM. We will also determine whether changes in cerebral pathophysiology (MRS, DTI-FW) correspond with changes in cognition, brain function (fMRI) and serum

inflammatory and liver biomarkers. In addition, we will determine which neuroimaging and baseline clinical factors are associated with long-term drinking and clinical outcomes (e.g. HIV viral suppression, liver comorbidities). In the context of this study, CM and MI are being employed as an experimental manipulation and data collection opportunity, respectively, rather than as clinical interventions per se. The A-B-A design will enable us to clearly identify whether alcohol effects on cognition and the brain are reversible, and to identify optimal strategies to promote short-term and long-term alcohol reduction in HIV+ adults. This U01 project is closely linked to the Administrative U24 (SHARC), which supports the Florida Cohort that is the source of potential participants for this study, and our Behavioral Science and Biostatistics Core (U24) that will help implement and monitor the CM, MI, and alcohol biosensor procedures.

PUBLIC HEALTH RELEVANCE

This study has strong public health relevance, because we will examine strategies to improve brain and cognitive function, which are among the most significant medical comorbidities associated with HIV and aging. The research will also help to identify improved strategies to help HIV+ persons reduce drinking.

CRITIQUE 1:

Significance: 3
Investigator(s): 3
Innovation: 1
Approach: 8
Environment: 1

Overall Impact:

This team proposes a project that will enroll 180 HIV+ adults with heavy drinking, and then use CM with financial incentives and a wearable alcohol biosensor to maximally reduce alcohol consumption from baseline (T1) to 4-weeks later (T2). They will then conduct a motivational interview to determine perceived benefits and obstacles to drinking reduction, and conduct a final assessment 1 year later (T3), at which point persons may or may not have resumed heavy drinking. They will conduct neuroimaging, cognitive, and behavioral assessments at each time point, and then continue to track long-term drinking and HIV outcomes in their companion cohort that is part of a separate U24. The investigators and the environment represents key strengths of this proposal, however, there are perhaps too many investigators with overlapping expertise. This application is innovative with the main idea of examining neuroimaging, cognitive, and behavioral assessments during a long-term follow-up visit after inducing abstinence. Unfortunately, there are several methodological weaknesses that reduce the significance and overall impact of this application. A major weakness is the length of the contingency management (CM) period. 4 weeks seems short, as most trials are between 12 and 16 weeks in length in order to get a large number of participants to achieve abstinence. It is unclear from the pilot data and previous literature whether such a short period of potential abstinence could produce better cognitive and brain function 1 year later. There are questions about the analytic plan and recruitment too. For example, there is no apparent plan to handle missing data, and the dropout rate is expected to be 10%, but there is no rationale given for this. Overall, this is a highly innovative potentially high impact project. The number of major methodological weaknesses, however, significantly reduce the impact of this project.

1. Significance:

Strengths

- The location of where this study is proposed speaks to the potential impact of this study were it to be successful.

- The gap that this project would fill is certainly significant with respect to cognitive function among an aging HIV+ population.
- The total length of observation for this study also speaks to impact of this project. This is especially true in light of being able to examine the reversibility.
- The comprehensiveness of the propose measures will also assist with making this a high impact project if successful.

Weaknesses

- The length of the CM intervention would significantly hinder the potential impact of this investigation.
- It unclear where the justification for a 1 year follow-up comes from in the presented literature.

2. Investigator(s):

Strengths

- The investigative team is well qualified to carry out the project.
- The Principal Investigators have a strong track record of carrying out work similar to this.

Weaknesses

- There appears to be significant overlap across site in expertise. There are 11 total investigators, which seems excessive in light of their expertise. It make sense that a study such as this will require several types of expertise, but this appears to be more than what is needed to be make the project successful.

3. Innovation:

Strengths

- The integration of several neuroimaging techniques is highly innovative. More generally, the overall comprehensiveness of the outcomes being examined is significantly innovative.
- The core idea of inducing alcohol abstinence and examining brain and cognitive improvements is highly innovative.
- The biosensor technology used in this trial is highly innovative.

Weaknesses

- None noted.

4. Approach:

Strengths

- Strong evidence of the team's ability to recruit this HIV+ population.
- The preliminary data demonstrating that alcohol use is associated with cognitive function helps to provide a compelling case for the current project.

Weaknesses

- 4 weeks is very short trial for CM and it may be difficult to find an effect. Is there a reason the investigators did not make the treatment period longer, e.g., 12 weeks which is consistent most previous CM trials? There is little rationale given for this (e.g., difficulty with recruitment), and thus, this represents a major weakness especially in the context of trying to examine both short-

term and long-term effects of alcohol abstinence/reduction. Is there any evidence that 4 weeks of abstinence will result in better brain and cognitive function 1 year later in any population, including an HIV+ population? If sustained abstinence or at least clinically significant reductions are not achieved or if this window of abstinence will not produce any kind of sustained improvement, the entire study will not succeed.

- There is no non-HIV group being compared because the literature has already demonstrated that HIV+ patients show worse brain and cognitive function. However, there is no indication as to whether HIV+ patients who abuse alcohol have been compared to an alcohol abusing patient population. Such would still be an important gap to fill and understand the relative, incremental benefit among these two populations.
- It is unclear exactly when the reinforce will be delivered to the participant. There is a note in the application about the importance of delivering the reinforce right away, and I would agree with that, but it is unclear when it takes place.
- There is some concern over a reset to $0 so that the participants receive nothing if they relapse. This could lead to significant dropout. One recommendation would be to set the level at $10 for simply providing data. There is also some concern over the ‘no reinforcement on a day when the biosensor malfunctions’. This could quickly increase dropout.
- There are several important details missing from the analytic plan. For example, how exactly will the random coefficient model be specified? How will missing data be handled? There is mention of longitudinal data, but it isn’t clear whether a random effect will be specified for time (this is addressed for Aim 2, not Aim 1)? How will potential site-effects be handled? This is important because this study includes the analysis of ‘change’ over time as the primary interest, rather than a treatment effect like one might expect in a study like this. Will the investigators be examining change between T1 and T2, then between T1 and T3? This is what was expected from the Aims but this doesn’t appear to be the core component of the planned analyses.
- The connection between the power analyses and what specific effects are being referenced in the preliminary data section is missing. At this point, it is unclear where these effects came from.
- It is unclear where the estimate of 10% dropout came from – this seems low for a project such as this.
- There is a statement about the expectations of 1/3 of participants will stop drinking, 1/3 will reduce a moderate amount, and 1/3 will reduce their drinking a little or not at all. Where does this categorization come from? Has this been found in previous CM 4-week interventions?

5. Environment:

Strengths

- The combined environments where the data will be collected are all very strong and will support this work.

Weaknesses

- None noted.

Protections for Human Subjects:

Acceptable Risks and/or Adequate Protections

- All risks have been adequately accounted for and managed appropriately.

Data and Safety Monitoring Plan (Applicable for Clinical Trials Only):

Acceptable

- A DSMP is not required because this is not a clinical trial, but the investigators still provided a DSMP.

Inclusion of Women, Minorities and Children:

- Sex/Gender: Distribution justified scientifically
- Race/Ethnicity: Distribution justified scientifically
- Inclusion/Exclusion of Children under 21: Excluding ages < 21 justified scientifically
- The inclusion criteria for women, minorities, and children is appropriate given the scope of this project

Vertebrate Animals:

- Not Applicable

Biohazards:

Not Applicable

Applications from Foreign Organizations:

- Not Applicable

Select Agents:

- Not Applicable

Resource Sharing Plans:

- Acceptable
- The resource sharing plan in adequate.

Budget and Period of Support:

Recommended budget modifications or possible overlap identified:

- As noted above, there appears to be too many investigators (11) and in some cases, there is significant overlap in expertise. Recommend examining the level of effort among the investigative team as this is the most expensive item on each of the budgets and likely does not need to be so high.

CRITIQUE 2:

Significance: 1
Investigator(s): 2
Innovation: 2
Approach: 3
Environment: 1

Overall Impact:

The proposed studies aim at expanding the Southern HIV Alcohol Research Consortium (SHARC) – diverse longitudinal HIV+ Florida cohort with a goal to examine the efficacy of decreasing alcohol consumption via a contingency management (CM) intervention paradigm. The studies are aimed at observing improvements in cognitive performance, brain functions and pathophysiology, and HIV-associated health outcomes. Importantly, the proposed studies have the potential to test the hypothesis that heavy alcohol mediated brain abnormalities in HIV+ individuals are reversible with interventions that significantly decrease alcohol use. The studies have clinical relevance, however, there are concerns regarding the relatively short tenure of 4 weeks of the CM intervention and its efficacy in improving cognitive and behavioral performance.

1. Significance:

Strengths

- Proposed studies examine a clinically significant hypothesis that alcohol-associated brain abnormalities in people with HIV may be reversible with a significant reduction in alcohol use.
- In pursuing the proposed hypothesis, the studies combine several cutting-edge neurocognitive and neuroimaging procedures with a variety of alcohol consumption reducing strategies via CM. Thus, leading to unique opportunities to examine the interactive effects of HIV and alcohol on the brain.

Weaknesses

- None noted

2. Investigator(s):

Strengths

- The PI and Co-PI, Drs. Cook and Cohen form an experienced team of investigators with considerable expertise in the area of HIV and alcohol that is highly relevant to pursue the proposed aims. They will be aptly supported by their Co-Investigators who are also well qualified in the proposed areas of research.

Weaknesses

- Budget Justification does not document the effort and contributions of other Co-PIs, Drs. Deviuex and Govind. Information related to these Co-PIs is also not included in the Multiple PI Leadership Plan.

3. Innovation:

Strengths

- Use of CM plan along with various strategies to reduce alcohol consumption in HIV+ individuals, with intent to changing drinking behavior and potentially reversing brain deficits is novel and innovative.
- Use and integration of neuroimaging techniques and procedures to assess improvement in brain health occurring in response to a reduction in alcohol consumption is innovative.
- Use of transdermal alcohol sensors to continuously monitor alcohol consumption and drinking patterns along with self-reported drinking is also innovative and significant for establishing objective measures of alcohol consumption in the future.

Weaknesses

- None noted.

4. Approach:

Strengths

- The proposed studies aimed at reducing health disparities in HIV-related outcomes employ a diverse cohort (in terms of gender and race/ethnicity) affected by alcohol and HIV infection and pursue longitudinal assessments.
- Tests the efficacy of a variety of previously tested interventional approaches aimed at reducing alcohol consumption in the context of a contingency management (CM) strategy which involves giving tangible rewards to reinforce positive behaviors such as abstinence.
- Combination of neurocognitive and neuroimaging procedures with alcohol reducing strategies that would allow the examination of the interactive effects of HIV and alcohol abuse on the brain pathophysiology.
- Examination of the association of changes in systemic inflammation with brain pathophysiology and function.
- Application of a study design that employs a pre-post comparison with extended follow-up that has the potential to address the question of reversibility of alcohol-induced brain deficits in HIV+ individuals.

Weaknesses

- Although abstinence allows an improvement of cognitive functions in alcohol abusing individuals, this is only achieved after a period of several months. Accordingly, the time of 4 weeks (T2) to determine the effectiveness of a contingency management that encourages alcohol cessation seems questionable/inadequate.
- It is not clear if alternative strategies are available if the interventional strategies do not yield significant neurocognitive therapeutic value.

5. Environment:

Strengths

- Technical and scientific resources and environment at the University of Florida is excellent.

Weaknesses

- None noted.

Protections for Human Subjects:

- Acceptable Risks and/or Adequate Protections

Inclusion of Women, Minorities and Children Applicable Only for Human Subjects Research:

G1A - Both Genders, Acceptable

M1A - Minority and Non-minority, Acceptable

C3A - No Children Included, Acceptable

Vertebrate Animals:

- Not Applicable

Biohazards:

- Acceptable

Renewal:

- This UO1 is a competitive renewal and is part of the previously established Southern HIV and Alcohol Research Consortium (SHARC).

Resource Sharing Plans:

- Acceptable

Budget and Period of Support:

- Recommend as Requested

CRITIQUE 3:

Significance: 4
Investigator(s): 2
Innovation: 4
Approach: 4
Environment: 2

Overall Impact:

The investigators plan to quantify cognitive performance and brain structure and function at baseline, following a monitored 1-month abstinence period, and after one year in heavy drinking HIV+ individuals. Novel aspects of this grant are the monitored 1-month abstinence period (using an alcohol monitor and contingency management, i.e., paying subjects to remain sober) and the quantification of free water as an index of neuroinflammation. However, quantification of MRS metabolites and fMRI metrics following 1-month of abstinence is not novel and not clear this aspect of the proposal will provide any new information.

1. Significance:

Strengths

- One of the strengths of this grant is the use of contingency management and the biosensors to promote and monitor abstinence over 1 month. This controlled abstinence is novel and significant for the kind of work proposed.
- This has the potential to inform and advance treatment strategies.

Weaknesses

- Not clear that MRS or fMRI experiments in this proposal will provide any novel information to the field.

2. Investigator(s):

Strengths

- The research team has a track record in dealing with HIV subjects.
- The research team has been productive with respect to publications.
- The consultant, Dr. Pasternak, developed DTI-FW as a method for quantifying free water in the brain as an index of neuroinflammation.

Weaknesses

- Concern about the effort required to track and contact subjects as frequently as intended (i.e., can RAs handle the work load?)

3. Innovation:

Strengths

- DTI-FW to quantify free water as an index of neuroinflammation is novel.
- Monitored abstinence is also novel (especially for non-in-patient alcohol recovery)

Weaknesses

- Not enough detail regarding DTI-FW acquisition and analysis. For example, what b-value shells will be used?
- Is Pasternak going to set up the DTI-FW imaging protocol? What about the DTI-FW data analysis?

4. Approach:

Strengths

- The planned correlations between blood markers (cytokines and liver biomarkers) and brain MRS metabolites should result in some interesting data.
- Many of the subjects may already be identified from their participation in the larger cohort

Weaknesses

- MRS with 4 weeks of abstinence is not novel (see work by G. Ende and D. Meyerhoff).
- Available evidence suggests that MRS does not provide sufficiently unique markers of disease.
- Why are planned metabolites to be quantified as ratios to creatinine and not referenced to water?
- GABA quantification is challenging – not clear that a typical PRESS sequence can measure GABA. Also, might be residual as elsewhere states the focus is on NAA, Cho, and mI (Aim 2, H2.1).
- fMRI with 4 weeks of abstinence is not novel (see work by G. Fein)
- How will inflammation due to alcoholism be distinguished from inflammation due to HIV (from results of both MRS and DTI-FW?)
- Appears that preliminary work included only 13 individuals using the SCRAM device- seems that scaling to 180 subjects is a big jump and may have low compliance?

- A lot of work is proposed for the research assistants (RA). Is it feasible to have RAs in contact with 180 subjects as frequently as proposed?
- Contacting subjects via text? Many individuals in this study population may not have cell phones.
- Cumulative inclusion enrollment report (pg. 156) appears to include only female subjects while elsewhere suggest only 25% of participants will be female.
- Also, accounting for only 10% attrition seems low (especially among heavy drinking HIV+ individuals).
- Grant application is not always very clear or detailed.
- In Abstract, it is stated: "Our past findings indicate that *current alcohol use* is more strongly associated with cognitive and brain dysfunction among HIV+ adults than lifetime consumption" but in Significance section, it is stated: "the relative importance of current alcohol consumption versus lifetime use on the brain and cognition has not yet been clearly delineated, especially among HIV+ adults --- which one is it?
- "Having produced the most evaluation to date of the ability of the SCRAM bracelet" produced the most…? Of the ability of the bracelet to do what?
- Fig 3 – what individuals were included in Group A vs. Group B?

5. Environment:

Strengths

- Track record of imaging and following up this study population.

Weaknesses

- None noted.

Protections for Human Subjects:

Unacceptable Risks and/or Inadequate Protections

- Female participants should undergo a urine screen to confirm negative pregnancy status prior to imaging.

Data and Safety Monitoring Plan (Applicable for Clinical Trials Only):

Not Applicable (No Clinical Trials)

Inclusion of Women, Minorities and Children:

- Sex/Gender: Distribution not justified scientifically
- Race/Ethnicity: Distribution not justified scientifically
- Inclusion/Exclusion of Children under 21:
- There are several discrepancies regarding planned enrollment that need to be clarified before this question can be accurately answered.

Vertebrate Animals:

- Not Applicable

Biohazards:
Not Applicable (No Biohazards)

Applications from Foreign Organizations:
- Not Applicable (No Foreign Organizations)

Select Agents:
- Not Applicable (No Select Agents)

Resource Sharing Plans:
- Acceptable

Budget and Period of Support:
- Recommend as Requested

THE FOLLOWING SECTIONS WERE PREPARED BY THE SCIENTIFIC REVIEW OFFICER TO SUMMARIZE THE OUTCOME OF DISCUSSIONS OF THE REVIEW COMMITTEE, OR REVIEWERS' WRITTEN CRITIQUES, ON THE FOLLOWING ISSUES:

PROTECTION OF HUMAN SUBJECTS (Resume): ACCEPTABLE

INCLUSION OF WOMEN PLAN (Resume): ACCEPTABLE

INCLUSION OF MINORITIES PLAN (Resume): ACCEPTABLE

INCLUSION OF CHILDREN PLAN (Resume): ACCEPTABLE

COMMITTEE BUDGET RECOMMENDATIONS: The budget was recommended as requested.

Footnotes for 2 U01 AA020797-06; PI Name: Cook, Robert L

NIH has modified its policy regarding the receipt of resubmissions (amended applications). See Guide Notice NOT-OD-14-074 at http://grants.nih.gov/grants/guide/notice-files/NOT-OD-14-074.html. The impact/priority score is calculated after discussion of an application by averaging the overall scores (1-9) given by all voting reviewers on the committee and multiplying by 10. The criterion scores are submitted prior to the meeting by the individual reviewers assigned to an application, and are not discussed specifically at the review meeting or calculated into the overall impact score. Some applications also receive a percentile ranking. For details on the review process, see http://grants.nih.gov/grants/peer_review_process.htm#scoring.

MEETING ROSTER
National Institute on Alcohol Abuse and Alcoholism Special Emphasis Panel

NATIONAL INSTITUTE ON ALCOHOL ABUSE AND ALCOHOLISM
CHAART Consortium RFA
ZAA1 DD (10)
04/27/2016 - 04/29/2016

CHAIRPERSON(S)

PARSONS, JEFFREY T, PHD
DISTINGUISHED PROFESSOR
DIRECTOR, CENTER FOR HIV/AIDS EDUCATIONAL STUDIES AND TRAINING (CHEST)
HUNTER COLLEGE AND THE GRADUATE CENTER
OF THE CITY UNIVERSITY OF NEW YORK
NEW YORK, NY 10065

MEMBERS

BAGBY, GREGORY JOHN, PHD
PROFESSOR
DEPARTMENT OF PHYSIOLOGY
LSU HEALTH SCIENCES CENTER
NEW ORLEANS, LA 70112-1393

BALACHOVA, TATIANA N, PHD
ASSOCIATE PROFESSOR
DEPARTMENT OF PEDIATRICS
CHILD STUDY CENTER
THE UNIVERSITY OF OKLAHOMA HEALTH SCIENCES CENTER
OKLAHOMA CITY, OK 73117

BALASUBRAMANIAN, RAJI, DSC
ASSOCIATE PROFESSOR
DIVISION OF BIOSTATISTICS AND EPIDEMIOLOGY
SCHOOL OF PUBLIC HEALTH AND HEALTH SCIENCES
UNIVERSITY OF MASSACHUSETTS
AMHERST, MA 01003

BARVE, SHIRISH S, PHD
PROFESSOR
DEPARTMENT OF MEDICINE
AND PHARMACOLOGY AND TOXICOLOGY
UNIVERSITY OF LOUISVILLE
LOUISVILLE, KY 40202

CLARK, URAINA S, PHD
DEPARTMENT OF NEUROLOGY
ICAHN SCHOOL OF MEDICINE AT MOUNT SINAI
NEW YORK, NY 10029

DAVIS, KELLY CUE, PHD
RESEARCH ASSOCIATE PROFESSOR
SCHOOL OF SOCIAL WORK
UNIVERSITY OF WASHINGTON
SEATTLE, WA 98105

DOWDY, DAVID WESLEY, MD, PHD
ASSISTANT PROFESSOR
DEPARTMENT OF EPIDEMIOLOGY
BLOOMBERG SCHOOL OF PUBLIC HEALTH
JOHNS HOPKINS UNIVERSITY
BALTIMORE, MD 21205

KALICHMAN, SETH C, PHD
PROFESSOR
DEPARTMENT OF PSYCHOLOGY
UNIVERSITY OF CONNECTICUT
STORRS, CT 06269

KERR, WILLIAM C, PHD
SENIOR SCIENTIST
ALCOHOL RESEARCH GROUP
PUBLIC HEALTH INSTITUTE
EMERYVILLE, CA 94608

KRAEMER, KEVIN L, MD
ASSOCIATE PROFESSOR
HEALTH POLICY AND MANAGEMENT
SCHOOL OF PUBLIC HEALTH
UNIVERSITY OF PITTSBURGH
PITTSBURGH, PA 15213

MCPHERSON, STERLING M., PHD
ASSISTANT PROFESSOR
COLLEGE OF NURSING PROGRAM OF
EXCELLENCE IN ADDICTIONS RESEARCH
WASHINGTON STATE UNIVERSITY
SPOKANE, WA 99210

O'DONNELL, MAX, MD
ASSISTANT PROFESSOR OF MEDICINE
DEPARTMENT OF MEDICINE AND EPIDEMIOLOGY
COLUMBIA UNIVERSITY
NEW YORK, NY 10461

PALFAI, TIBOR P., PHD
ASSOCIATE PROFESSOR
DEPARTMENT OF PSYCHOLOGY
BOSTON UNIVERSITY
BOSTON, MA 02215

SILVERBERG, MICHAEL J, PHD
RESEARCH SCIENTIST
DIVISION OF RESEARCH
KAISER PERMANENTE
OAKLAND, CA 94612

ZAHR, NATALIE M, PHD
RESEARCH SCIENTIST
DEPARTMENT OF HEALTH SCIENCES
SRI INTERNATIONAL
MENLO PARK, CA 94025

SCIENTIFIC REVIEW OFFICER

SRINIVAS, RANGA, PHD
CHIEF, EXTRAMURAL PROJECT REVIEW BRANCH
EXTRAMURAL PROJECT REVIEW BRANCH
NATIONAL INSTITUTE ON ALCOHOL ABUSE AND ALCOHOLISM
NATIONAL INSTITUTES OF HEALTH
ROCKVILLE, MD 20855

EXTRAMURAL SUPPORT ASSISTANT

FULTON, THELMA
EXTRAMURAL SUPPORT ASSISTANT
OFFICE OF EXTRAMURAL ACTIVITIES
NATIONAL INSTITUTE ON ALCOHOL ABUSE AND ALCOHOLISM
NATIONAL INSTITUTES OF HEALTH
ROCKVILLE, MD 20892-9304

Consultants are required to absent themselves from the room during the review of any application if their presence would constitute or appear to constitute a conflict of interest.
